# Machine learning–assisted triboelectric nanogenerator technology for intelligent sports

**DOI:** 10.1126/sciadv.adz3515

**Published:** 2025-10-01

**Authors:** Minglan Ji, Zhen Wang, Jiamin Wu, Lijun Huang, Mingli Zheng, Gang Cheng, Huaihong Cai, Jianjun Luo, Haibo Zhou, Zhong Lin Wang

**Affiliations:** ^1^ CAS Center for Excellence in Nanoscience, Beijing Institute of Nanoenergy and Nanosystems, Chinese Academy of Sciences, Beijing 101400, P. R. China.; ^2^ School of Nanoscience and Engineering, University of Chinese Academy of Sciences, Beijing 100049, P. R. China.; ^3^ Key Lab for Special Functional Materials, Ministry of Education, National & Local Joint Engineering Research Center for High-efficiency Display and Lighting Technology, School of Nanoscience and Materials Engineering, and Collaborative Innovation Center of Nano Functional Materials and Applications, Henan University, Kaifeng 475004, P. R. China.; ^4^ Guangdong Second Provincial General Hospital, Postdoctoral Research Station of Traditional Chinese Medicine, College of Pharmacy, Jinan University, Guangzhou 510632, P. R. China.; ^5^ College of Chemistry and Materials Science, Jinan University, Guangzhou 510632, P. R. China.; ^6^ Guangzhou Institute of Blue Energy, Knowledge City, Huangpu District, Guangzhou 510555, P. R. China.

## Abstract

The rapid development of internet of things, big data, and artificial intelligence is propelling sports science into a data-driven era, demanding real-time, multidimensional athletic performance monitoring. Triboelectric nanogenerators (TENGs) have demonstrated exceptional potential in intelligent sports. However, the complexity and volume of TENG-generated data pose challenges for manual analysis. Machine learning (ML), with strengths in pattern recognition and adaptive processing, provides a powerful solution to enhance TENG-based sensing signal interpretation. This review systematically explores the integration of ML and TENG technology for intelligent sports. First, the fundamental theory and basic knowledge of TENGs are introduced, highlighting their versatility in sports sensing systems. Subsequently, a comprehensive overview of ML models for TENG signal analysis is discussed. Recent advancements of ML-assisted TENG-based intelligent sports applications, including sports training evaluation, sports health monitoring, and virtual/augmented reality sports, are then highlighted. Last, current challenges and future prospects of TENG-based intelligent sports systems are discussed.

## Teaser

Machine learning enhances triboelectric nanogenerator technology for intelligent sports systems.

## INTRODUCTION

The rapid advancement of the internet of things (IoT), artificial intelligence (AI), and big data has revolutionized the sports industry over the past decades, ushering it into the digital era (*1*). Data acquisition and analysis play a pivotal role in the development of intelligent sports, supporting enhanced athletic performance, injury prevention, and optimized training regimens. Real-time and accurate data acquisition relies heavily on distributed sensors, which are widely used in intelligent sports and use various detection mechanisms, including optical (*2*), capacitive (*3*), resistive (*4*), thermal (*5*), and electromagnetic (*6*) types. Despite their high sensitivity and versatility, traditional sensing technologies typically depend on external power supplies, resulting in limited operational lifespans, high replacement costs, frequent maintenance requirements, and potential environmental pollution. These limitations have substantially hindered the widespread adoption of smart sensing systems in sports applications. Consequently, the development of sustainable and maintenance-free sensing technologies is urgently needed to support long-term, real-time sports data monitoring.

In 2012, Z. L. Wang introduced the concept of the triboelectric nanogenerator (TENG) based on the coupling effect of friction initiation and electrostatic induction (*7*–*10*). TENGs can efficiently convert irregular, distributed, low-frequency mechanical energy into electrical energy such as human motion (*11*–*15*), vibration (*16*, *17*), ocean waves (*18*–*22*), and wind (*23*–*27*), demonstrating strong energy-harvesting capabilities. In addition, recent studies have demonstrated that TENGs can also harvest high-frequency mechanical energy such as ultrasound (*28*–*30*), further expanding their application in biomedical engineering and industrial ultrasonics. Moreover, by directly transforming mechanical stimuli into electrical signals, TENGs can also operate as self-powered sensors for pressure, acceleration, strain, or motion sensing without external power supply, which is vital for developing maintenance-free systems (*31*–*34*). Consequently, the TENG technology can be an effective solution for developing maintenance-free, distributed sensing systems in intelligent sports applications. However, the raw output signals produced by TENG-based sensing systems typically exhibit nonlinear characteristics, substantial noise interference, and sparse semantic information, complicating direct interpretation and limiting immediate applicability. Therefore, advanced data processing methodologies are required to fully exploit the potential of TENG technology in intelligent sports.

**Fig. 1.**
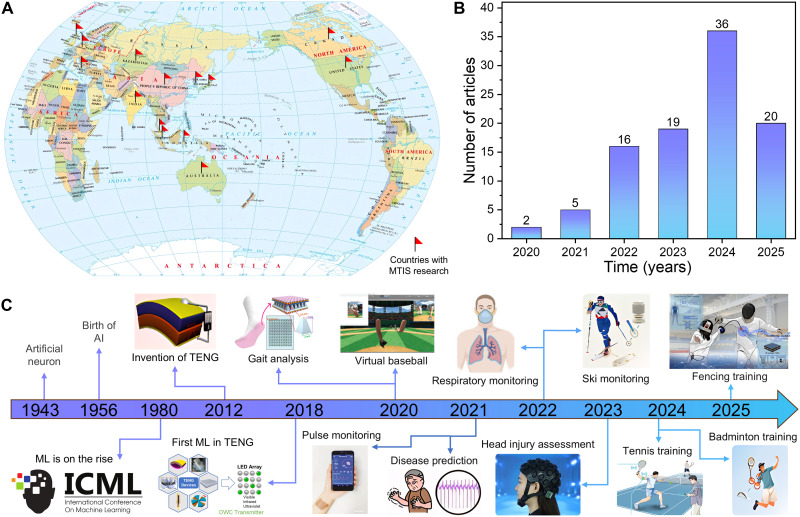
Literature survey in the field of intelligent sports with ML-assisted TENG technology from the SCI database by 30 April 2025. (**A**) MTIS application research conducted by universities and research institutes globally. (**B**) Number of MTIS research articles published each year. (**C**) Historical timeline and milestones in the field of MTIS. Image credits: Reused with permission from (*7*, *42*, *43*, *45*, *47*, *142*, *148*, *155*, *158*) and from (*48*, *143*, *161*) (CC BY for all references; https://creativecommons.org/licenses/by/4.0/deed.en).

In 1943, McCulloch and Pitts proposed the first “artificial neuron” model, laying the theoretical foundation for the development of neural networks (*35*, *36*). In 1956, MacCarthy and colleagues organized a conference called “Dartmouth Summer Research Project on Artificial Intelligence,” during which the term AI was formally introduced, marking the birth of the AI field (*37*). The subsequent rise of machine learning (ML) was marked by the inaugural International Conference on Machine Learning held in 1980 (*38*, *39*). As a powerful tool for data-driven analysis, ML enables intelligent decision-making, prediction, and adaptive responses by identifying complex patterns within large datasets (*40*). Its ability to process vast and intricate information makes ML highly suitable for enhancing the interpretability and utility of TENG-based sensing data (*41*). Recent advancements in ML algorithms and frameworks have further amplified the capability to extract meaningful insights from complex TENG outputs, enabling accurate motion analysis, precise pattern recognition, and improved environmental adaptability for sports applications. In 2018, Ding *et al.* (*42*) introduced the integration of TENG technology and ML algorithms, combining self-powered sensing with AI-driven data processing. This interdisciplinary fusion has propelled the development of intelligent sports, enabling real-time monitoring, personalized training, and precise assessment. In recent years, ML-assisted TENG systems have been increasingly applied across domains such as sports training (*43*, *44*), health monitoring (*45*–*47*), and virtual/augmented reality (VR/AR) sports (*48*–*50*), demonstrating their strong potential to reshape athletic performance and user experiences. According to literature analyses, the adoption of TENG and ML technologies in intelligent sports spans over 14 countries and regions (Fig. 1A), with the number of related publications increasing rapidly each year (Fig. 1B), reflecting the global recognition and rapid development of this research area. The convergence of ML and TENG has driven substantial advancements in ML-assisted TENG-based intelligent sports (MTIS), marked by key milestones and innovative applications (Fig. 1C).

**Fig. 2.**
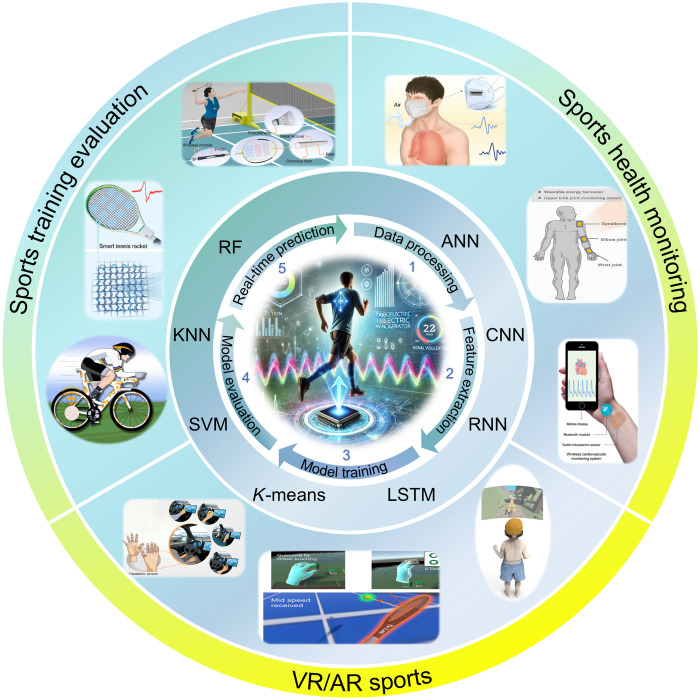
ML-assisted TENG technology for intelligent sports applications, including steps of ML in TENG, major ML models, and three MTIS applications. Sports training evaluation image credits: Reused with permission from (*145*, *147*, *148*). Sports health monitoring image credits: Reused with permission from (*47*, *120*, *151*). VR/AR sports image credits: Reused with permission from (*162*, *170*) and from (*48*) (CC BY; https://creativecommons.org/licenses/by/4.0/deed.en).

The increasing demand for personalized and data-driven sports technologies has positioned the integration of TENGs with ML as a transformative approach for advancing intelligent sports systems. Although previous reviews have explored various TENG-based wearable devices and sports equipment, they often lack a focus on generalized and adaptable ML algorithms necessary to fully harness the potential of TENG-based intelligent sports systems (*51*–*53*). In contrast, this review provides a systematic and comprehensive analysis of recent progress in MTIS research. We particularly emphasize how the synergistic interplay between TENG-based self-powered sensors and ML-based data analysis enables advanced capabilities such as enhanced motion recognition, adaptive feedback, real-time motion sensing, and data-driven decision-making, thereby expanding application domains (*54*, *55*). The structure of this review is organized into four main sections, as illustrated in Fig. 2. First, the fundamental mechanisms, first-principle theory, and working modes of TENGs are introduced. Second, an overview of commonly used ML and deep learning (DL) models relevant to TENG data processing is presented. Subsequently, the recent progress of MTIS applications is highlighted, including sports training evaluation, sports health monitoring, and VR/AR sports. Last, we discuss current challenges, emerging trends, and future opportunities for interdisciplinary development in this evolving field. By synthesizing existing state-of-the-art research, this review aims to offer both theoretical insight and practical guidance for the next generation of intelligent, self-powered sports technologies.

## TENG

Theoretical research forms the cornerstone of a scientific discipline. In the field of TENG, fundamental studies have played a particularly pivotal role in transforming empirical observations into quantifiable physical principles. Over the past few years, researchers have made substantial progress in the fundamental physics of the TENG. This section provides a systematic review of the latest progress in both the underlying principles and practical implementations of this research area.

### Mechanism of contact electrification

TENG generally consists of two dielectric materials with distinct triboelectric properties. When these materials undergo periodic contact and separation, charge transfer occurs at their interface, resulting in the generation of electricity that can be either stored or directly used to power electronic devices (*56*, *57*). Through the coupling effect of contact electrification and electrostatic induction, mechanical energy is efficiently converted into electrical energy (*58*–*61*). To explain the mechanism of contact electrification (*62*, *63*), Wang and Wang (*8*) proposed the electron-cloud overlap model (fig. S1, A and B). In this model, electrons on the surfaces of the materials are initially confined within potential wells. Upon contact, electron clouds of the two materials overlap, causing electron redistribution and facilitating charge transfer. During the subsequent separation, potential barriers hinder electron recombination, leading to charge retention on the material surfaces and the formation of an electric field. On the basis of this fundamental charge transfer process, we use the contact-separation mode as an example to further describe how TENG operates dynamically (fig. S1C). Initially, no charge is generated or transferred (fig. S1C, i). When two different materials come into contact under an externally applied force, contact electrification occurs, leading to the generation of opposite triboelectric charges on the surfaces of the materials (fig. S1C, ii). As the applied force decreases, the materials begin to separate. This separation results in the formation of an electric potential difference between the electrodes, which drives a charge flow through an external circuit (fig. S1C, iii). Last, the materials are fully separated, and a new equilibrium state is established (fig. S1C, iv). When the surfaces are pressed again, charges flow back through the load to offset the potential difference (fig. S1C, v). This cycle can be continuously repeated, enabling the generation of alternating current power.

### First-principle theory of TENGs

From 2021 to 2025, the theory describing the electromagnetic phenomena in electromagnetic mechanosystems has been developed for quantifying the output of TENG. Wang (*64*) derived Maxwell’s equations for a mechano-driven medium system (MEs-f-MDMS) under low-speed approximation (*v* << *c*). The MEs-f-MDMSs are essential for describing the electrodynamics of a moving object that undergoes not only accelerated translational motion but also rotational motion. In a system composed of moving media or objects, the electromagnetic behavior is described in the Lab frame (S frame), while the moving object is present in the space that is traveling with its reference center *S*′ at v(t) . The electromagnetic behavior inside the moving medium is described by the MEs-f-MDMS equations, which are given by

∇·D=ρ (1a)

∇·B=0 (1b)

$$\nabla \times \left(E+v_{r}\times B\right)=-\frac{\partial }{\partial t}B$$ (1c)

$$\nabla \times \left(H-v_{r}\times D\right)=J+\rho v+\frac{\partial }{\partial t}D$$ (1d)

where v(t) is the translation moving velocity of the origin of the reference frame $S^{\prime }$ , which is only time-dependent, and $v_{r}\left(r,t\right)$ is the relative velocity of the point charge with respect to the reference frame $S^{\prime }$ , which is space- and time-dependent, and can be simply referred as “rotation speed.” The classic Maxwell’s equations are to describe electrodynamics in the region where there is no local medium movement. The full solutions of the two regions satisfy the boundary conditions so that the rotation of the object affects the electromagnetic field at vicinity.

In practical engineering, there are many cases that have no translation motion but rotation motion of the object, as presented in this section. In such case, we have v=0 so that $v_{t}\left(r,t\right)$ = $v_{r}\left(r,t\right)$ . This is for the case that not only the system can rotate around the origin, but also its surface and volume as well as boundaries can experience expansion and/or contraction. Using the constitutive relationship, Eqs. 1a and 1b reduce to (*65*)

$$\varepsilon \nabla \cdot E_{\text{eff}}=\;\rho$$ (2a)

$$\mu \nabla \cdot H_{\text{eff}}=\;0$$ (2b)

$$\nabla \times E_{\text{eff}}\approx -\mu \frac{\partial }{\partial t}H_{\text{eff}}$$ (2c)

$$\nabla \times H_{\text{eff}}\approx J+\varepsilon \frac{\partial }{\partial t}E_{\text{eff}}$$ (2d)

where the effective fields are defined by

$$E_{\text{eff}}=E+\mu \;v_{r}\times H$$ (3a)

$$H_{\text{eff}}=H-\varepsilon \;v_{r}\times E$$ (3b)

Equations 2a to 2d are identical to the classical Maxwell equations, except that the electric field E and magnetic field H are replaced by the effective fields $E_{\text{eff}}$ and $H_{\text{eff}}$ , respectively. In Eq. 3a, the second term on the right-hand side is the Lorentz force. Similarly, in Eq. 3b, the second term is the contribution of the local electric field to the local magnetic field because of medium movement. Therefore, the medium motion is a source for generating electromagnetic fields, the motion of a medium in the presence of a magnetic field generates an additional component to the electric field, and the medium motion in the presence of an electric field would generate an additional term to the magnetic field. The propagation, scattering, and reflection of the effective fields satisfy the classical Maxwell equations as for the case where there would be no medium motion. Such equations are the first-principle theory for quantifying the output of TENG and related systems.

### Working modes of TENGs

**Fig. 3.**
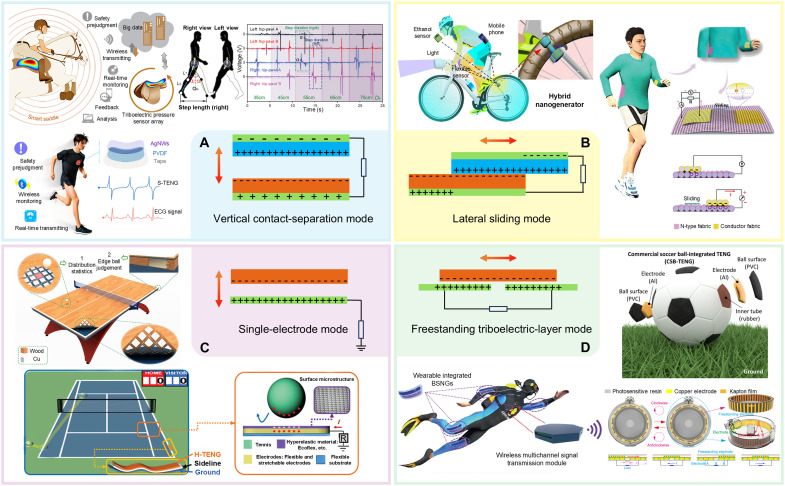
Four fundamental working modes of TENG. (**A**) Vertical CS mode in intelligent sports. Image credits: Reused with permission from: (*67*, *73*) and from (*188*) (CC BY; https://creativecommons.org/licenses/by/4.0/deed.en). (**B**) Linear sliding mode in intelligent sports. Image credits: Reused with permission from: (*80*, *189*). (**C**) SE mode in intelligent sports. Image credits: Reused with permission from: (*95*) and from (*83*) (CC BY; https://creativecommons.org/licenses/by/4.0/deed.en). (**D**) FT mode in intelligent sports. Image credits: Reused with permission from: (*86*) and from (*90*, *91*) (CC BY; https://creativecommons.org/licenses/by/4.0/deed.en).

The operation of TENGs can be classified into four fundamental working modes on the basis of polarization behavior and electrode configuration: vertical contact-separation (CS) mode, lateral sliding (LS) mode, single-electrode (SE) mode, and freestanding triboelectric-layer (FT) mode, as illustrated in Fig. 3. As the most basic structure, the vertical CS mode uses the relative movement perpendicular to the interface. During the CS process, the potential changes between the two electrodes and an external current flow will then be created for balancing the potential difference. Given its high sensitivity to mechanical pressure, this mode is particularly suited for applications such as haptic electronic skin for pressure detection (*66*, *67*), which can be used in sports to monitor movement patterns (*68*–*70*), analyze gait (*71*, *72*), and measure impact forces (*73*, *74*) during training. Its precise pressure feedback capability allows for the enhanced assessment of athletic performance and postural stability (*75*–*78*). In LS mode, the lateral displacement between contact surfaces induces charge transfer and creates a continuous potential difference. This configuration is highly responsive to dynamic movements (*79*), making it ideal for real-time motion tracking. For instance, LS mode can be used in motion analysis systems to assess stride length, joint movement, and muscle fatigue during activities such as running and cycling (*80*, *81*), offering valuable insights into an athlete’s biomechanics and endurance. SE mode, which relies on charge generation through direct contact with a moving object, eliminating the need for a paired electrode. Its simple structure and lightweight design make it particularly suitable for integration into flexible and wearable electronics (*82*). This adaptability allows SE-mode TENGs to be embedded in sportswear or equipment for monitoring both high- and low-frequency movements (*83*, *84*), enabling self-powered sensing without compromising wearer comfort (*13*, *85*). The FT mode builds upon the SE mode by introducing a freely movable friction layer between two electrodes (*86*, *87*). This configuration enhances structural flexibility and allows integration into textiles, smart flooring (*88*), footballs, and face masks (*89*), enabling nonintrusive, real-time monitoring for both professional athletes and general users (*90*, *91*).

The versatility of these four TENG working modes supports diverse applications in intelligent sports, addressing the critical requirements of real-time monitoring, long-term durability, and self-powered operation. By selecting appropriate materials and configurations, TENG-based sensors can be seamlessly embedded into wearable systems and training equipment, ensuring both athlete comfort and accurate data collection. To date, the TENG technology has been successfully applied in a wide range of sports disciplines, including badminton (*92*), volleyball, basketball (*93*), baseball (*94*), soccer (*86*), tennis (*95*, *96*), ping-pong (*97*), golf (*98*), long jump (*99*), skating (*100*), boxing (*101*), Taekwondo (*102*), wheelchair sports (*103*), riding (*73*), yoga (*104*), and fencing (*105*). This integration into intelligent sports systems holds substantial promise for advancing performance analytics, personalized health management, and injury prevention, paving the way for more efficient and data-driven athletic development. In addition to the TENG technology, we have compared various sensing technologies for sports monitoring on the basis of different mechanisms, including resistive, capacitive, electromagnetic, optical, piezoelectric, and pyroelectric, as summarized in table S1. Compared with these technologies, TENGs exhibit unique advantages in sports scenarios, such as high efficiency under low-frequency motion, light weight, broad material selection, and self-powered operation, making them particularly suitable for real-time motion monitoring in dynamic athletic environments.

## ML FOR INTELLIGENT SPORTS

**Fig. 4.**
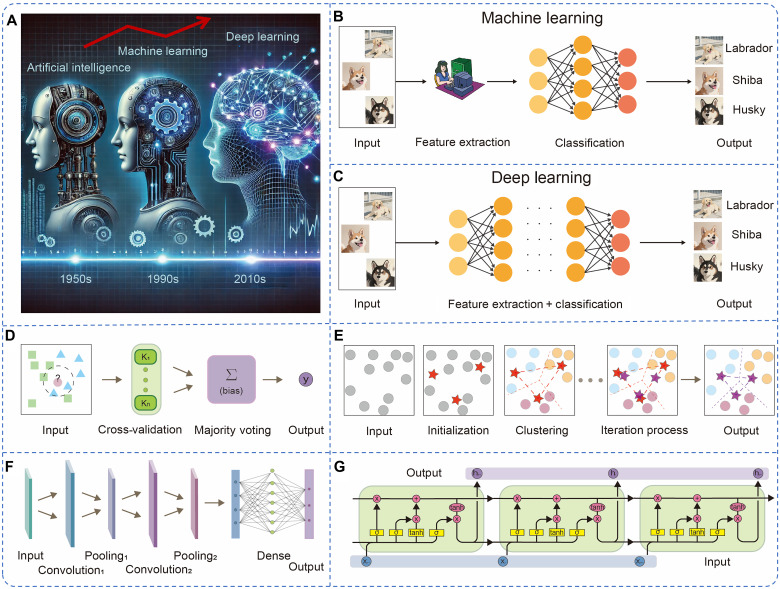
Evolution and major models of ML. (**A**) Relationship between AI, ML, and DL (*190*). (**B**) Data processing for ML. (**C**) Data processing for DL. (**D**) Typical structure of KNN (*191*). (**E**) Typical structure of *K*-means (*192*). (**F**) Typical structure of CNN (*193*). (**G**) Typical structure of LSTM (*194*).

With the exponential growth of the IoT, interconnected devices and sensor networks continuously generate vast amounts of real-time data. Effectively processing and analyzing these data have become a critical challenge, necessitating more advanced computational approaches. Consequently, AI, particularly ML, has gained considerable attention for its ability to extract meaningful insights from complex datasets, enabling predictive analysis and decision-making (Fig. 4A). ML models can be generally categorized into traditional ML and DL. Traditional ML includes supervised and unsupervised learning models, which often rely on handcrafted features and domain expertise for optimal performance (Fig. 4B). While effective in certain contexts, this reliance on manual feature engineering can limit model adaptability and scalability. In contrast, DL, a subset of ML, leverages advanced neural network architectures to automatically extract and refine features from large-scale datasets. By learning hierarchical patterns within data, DL substantially enhances tasks such as classification, prediction, and information retrieval, all without requiring explicit human intervention (Fig. 4C). This autonomous feature learning capability has led to the rise of large language models such as ChatGPT and DeepSeek, which are built on DL architectures. These models allow users to input queries through simple interfaces and receive accurate, context-aware responses, reducing the need for deep technical knowledge of the underlying systems. As AI models continue to evolve, their integration with IoT infrastructure and big data platforms is expected to revolutionize a wide range of fields from intelligent automation to personalized decision support systems. This section provides an overview of key ML models, emphasizing their working mechanisms and comparative advantages in intelligent data processing.

### Traditional ML models

ML models are generally classified into supervised and unsupervised learning, each exhibiting distinct mechanisms and application scenarios. Common algorithms include random forest (RF) (*106*), support vector machine (SVM) (*107*), *K*-nearest neighbors (KNN) (*108*), and *K*-means clustering (*109*).

Supervised learning involves training models on labeled datasets to establish a functional mapping between input features and target outputs. This approach enables accurate predictions on new, unseen data and is typically used for classification and regression tasks. Among the commonly used supervised algorithms, RF, SVM, and KNN are widely adopted. RF enhances accuracy and robustness by aggregating multiple decision trees, although it can be computationally intensive. SVM is particularly effective for small sample sizes and high-dimensional data using optimal hyperplanes for classification, but its performance diminishes with larger datasets because of increased computational demands. KNN, a simple and nonparametric method, classifies or predicts outcomes on the basis of proximity to neighboring data points. While intuitive, KNN is sensitive to noise and incurs high inference costs (Fig. 4D).

In contrast, unsupervised learning does not rely on labeled data but instead focuses on uncovering hidden patterns or structures within the dataset. A key task in this domain is clustering, with *K*-means being one of the most widely used algorithms (Fig. 4E). *K*-means partitions the data into a predefined number of clusters by minimizing intracluster variance. It works through an iterative process of selecting initial cluster centers, assigning data points to the nearest cluster, and updating the centroids until convergence. Although computationally efficient, *K*-means is sensitive to initial centroid selection and may perform poorly with nonspherical or imbalanced clusters. Despite these challenges, its simplicity and scalability make it a valuable tool in exploratory data analysis and pattern recognition.

### DL models

DL is a subset of ML that simulates the neural processing mechanisms of the human brain. These models use multilayered artificial neural networks (ANNs) to process complex and high-dimensional data structures, enabling the automatic extraction of hierarchical features. Common DL architectures include the ANN (*110*), convolutional neural network (CNN) (*111*), recurrent neural network (RNN) (*112*), and long short-term memory (LSTM) (*113*).

The ANN is the most fundamental DL model, consisting of an input layer, hidden as an output layer. Hidden layers transform features through weighted connections and activation functions, enabling the model to learn complex patterns. The CNN (Fig. 4F) excels at handling images of varying sizes, demonstrating exceptional performance in tasks involving image and video recognition as well as classification. CNNs use convolutional layers to extract spatial features and pooling layers to reduce dimensionality, enhancing computational efficiency and generalization performance. The RNN is designed for sequential data analysis, such as speech recognition and time-series forecasting. RNNs store historical information through recurrent connections but suffer from the vanishing gradient problem, limiting their ability to capture long-term dependencies. LSTM (Fig. 4G) is an advanced form of RNN that introduces memory cells and gating mechanisms (input, forget, and output gates) to regulate information flow. LSTMs effectively handle long-range dependencies, making them widely used in natural language processing and sequence-based prediction tasks.

In summary, each ML model has unique advantages and limitations. Traditional models such as RF, SVM, and KNN perform well on structured data and clearly defined tasks, while unsupervised models like *K*-means are valuable for uncovering hidden patterns in unlabeled data. DL models such as the ANN, CNN, RNN, and LSTM are highly effective for processing complex, high-dimensional data but require substantial computational resources. Therefore, selecting an appropriate model depends on the nature of the dataset, task requirements, and available computational capacity.

### ML-enhanced TENG signal analysis and model selection

The integration of ML with TENGs provides a transformative solution for intelligent sports applications, particularly in addressing the challenges of real-time data acquisition, interpretation, and feedback. TENG-based self-powered sensors enable the effective collection of athletes’ kinematic parameters [e.g., gait (*114*, *115*), knee angle (*116*), and impact (*117*)] and physiological metrics [e.g., heart rate (*47*), blood pressure (*118*, *119*), and respiratory rate (*120*, *121*)]. Subsequent ML-driven processing enhances data reliability through automated cleaning, feature extraction, and standardization while uncovering hidden biomechanical patterns and potential health risks for performance optimization.

**Fig. 5.**
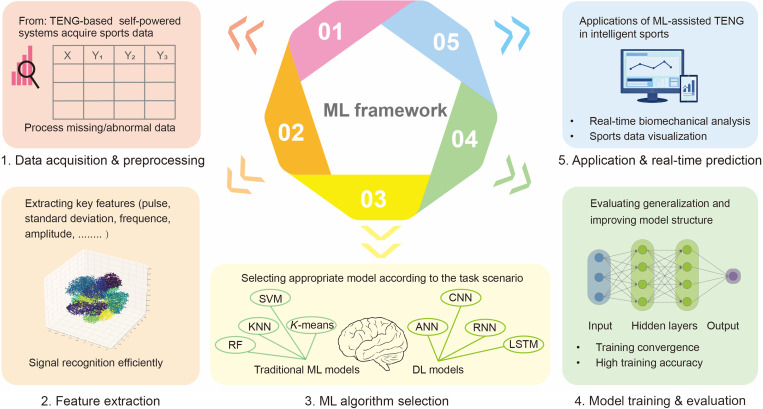
General framework of ML in TENG-generated sports data processing.

To fully harness this synergy, a systematic and standardized data processing framework is essential. Accordingly, a structured five-stage pipeline is typically used to ensure the robust analysis of TENG-generated data, as illustrated in Fig. 5. (i) Data acquisition and preprocessing: The distributed TENG-based self-powered sensor network captures high-resolution motion data, transmitting it to a centralized processing unit. Initial classification preprocessing involves outlier rejection and signal smoothing to improve the signal-to-noise ratio. (ii) Feature extraction and normalization: Key features (e.g., pressure distribution, gait period, and impact frequency) are algorithmically extracted from the raw data and normalized to conform to ML input specifications. (iii) ML algorithm selection: Appropriate ML algorithms, such as the SVM, RF, and CNN, are selected on the basis of data characteristics and task requirements, with the emphasis on predictive performance, scalability, and model interpretability. (iv) Model training and evaluation: The dataset is partitioned into training (80%) and testing (20%) subsets in most experiments. During training, hyperparameter optimization is performed to maximize model accuracy through techniques, such as Grid search or Bayesian optimization. Last, model performance is evaluated on the test set using appropriate metrics, including the confusion matrix and convergence analysis. (v) Real-time prediction and feedback: The trained model is implemented within the TENG-based self-powered intelligent system, enabling real-time biomechanical monitoring and actionable feedback for sports performance and health management.

Selecting the appropriate ML model is critical for optimizing TENG-based motion analysis, as different algorithms exhibit unique advantages in pattern recognition, classification, and prediction tasks (Table 1). Among ML approaches, supervised learning models, which rely on labeled data, are particularly effective for real-time motion classification and performance evaluation. For instance, RF leverages multiple decision trees to improve prediction accuracy (*122*, *123*), making it suitable for gait pattern analysis and athletic performance assessment. The KNN classifies motion types on the basis of distance metrics and is applicable to stride length, cadence, and other movement parameters (*124*). The SVM excels in small-sample, high-dimensional data analysis and is effective for monitoring physiological indicators such as heart rate and fatigue (*125*).

**Table 1.** Commonly used ML algorithms and their performance characteristics.

	Model	Key characteristics	Advantages	Disadvantages
Traditional machine learning (*197*, *198*)	RF	Ensemble of decision trees	Efficient, handles nonlinear relationships	High computation, poor interpretability
	KNN	Distance-based classifier	Simple, no training needed	Slow inference, sensitive to noise
	SVM	Hyperplane-based classifier	Effective for small, high-dimensional datasets	Complex tuning, slow on large datasets
	*K*-means	Unsupervised learning algorithms	Efficient, scalable	Requires *K*, sensitive to initialization
Deep learning (*199*)	ANN	Multilayer neural network	Captures complex patterns, automatic feature learning	Overfitting, long training time
	CNN	Spatial data processor	Effective for multiple array data	High computation, large data requirements
	RNN	Sequential data model	Learns time-dependent features	Gradient issues, high computation
	LSTM	Memory-enhanced RNN	Captures long-term dependencies	High computation

In contrast, unsupervised learning is useful for pattern discovery and data clustering. *K*-means clustering aids in categorizing movement styles and detecting anomalies, contributing to personalized training and injury prevention, while the ANN extracts complex motion features, proving valuable in motion recognition and prediction (*126*, *127*). DL further enhances feature extraction and time-series modeling capabilities, making it highly effective in TENG-based sports monitoring. CNNs, well suited for spatial data processing, enable accurate motion posture recognition and biomechanical analysis (*128*–*131*). RNNs, with their sequential modeling ability, are ideal for step counting and force detection (*132*–*134*), whereas LSTM networks mitigate long-term dependency issues, making them highly effective for gait cycle analysis (*135*, *136*), fatigue prediction, and impact force assessment.

By leveraging techniques such as data augmentation, hyperparameter optimization, and model fusion (e.g., XGBoost integrating RF, SVM, and LSTM), the ML-assisted TENG system can efficiently analyze motion data, improving the accuracy and reliability of intelligent sports monitoring (*137*–*140*). This integration provides robust data support for personalized training and health management, ultimately enhancing athletic performance and injury prevention.

## APPLICATIONS OF MTIS

To meet the growing demands of intelligent sports monitoring, the TENG-based sensing technology has undergone substantial advancements through material innovation and structural optimization. These developments have enabled their widespread implementation in wearable devices, smart equipment, and intelligent sports venues. Modern TENG-based self-powered sensors now incorporate lightweight, durable, and biocompatible materials with enhanced waterproofing capabilities, enabling high-precision signal acquisition for comprehensive motion tracking and physiological monitoring. When combined with ML, these self-powered sensors facilitate efficient data analysis and provide accurate assessments of both kinematic and physiological parameters. Furthermore, the incorporation of edge computing and adaptive algorithms supports low-latency, decentralized applications, enabling real-time data processing. These technological advancements have propelled the development of ML-assisted TENG systems for intelligent sports applications, with notable impact in three key areas: (i) sports training evaluation, enabling data-driven optimization of athletic techniques; (ii) sports health monitoring, providing real-time physiological assessment and injury prevention; (iii) VR/AR sports, making exercise more interesting. These innovations not only enhance the effectiveness of intelligent sports systems but also lay the foundation for scalable implementations in personalized health care and the next generation of smart sports ecosystems.

### Sports training evaluation

**Fig. 6.**
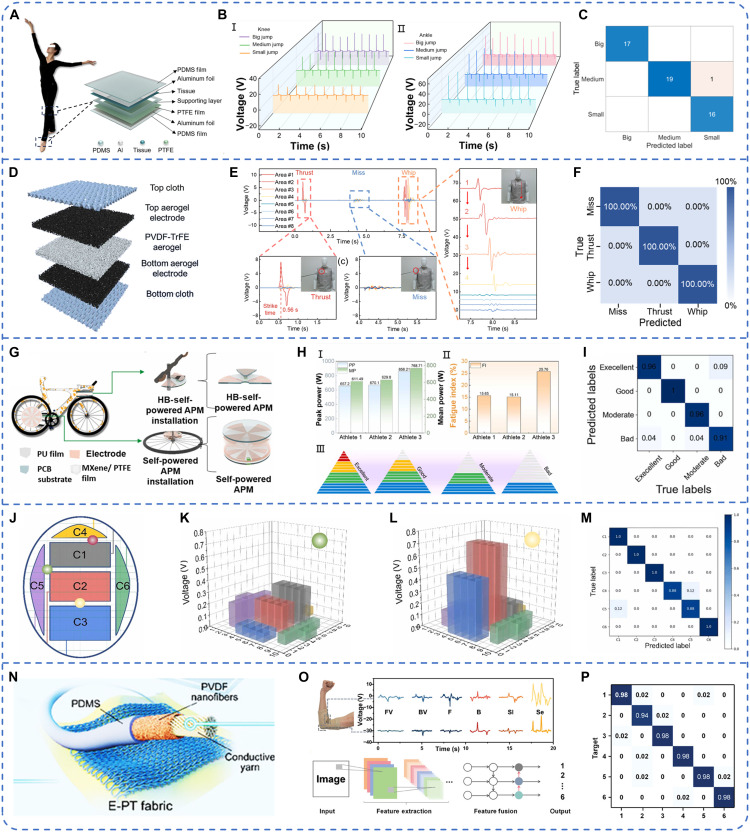
ML-assisted TENG for sports training evaluation. (**A**) Concept diagram of the dance sports injury monitoring system and RF-TENG structure. (**B**) Dance sports and injury monitoring system for dance ground-jumping technique monitoring. (**C**) Confusion matrix of test results. Image credit: (*141*) (CC BY; https://creativecommons.org/licenses/by/4.0/deed.en). (**D**) Schematic diagram of the structure design of the all-aerogel TENG. (**E**) Real-time signals from smart fencing cloth under the different hit patterns. (**F**) Confusion matrix of test results. Image credit: Reused with permission from (*142*). (**G**) Component part of APM. (**H**) Athlete selection system. (**I**) Accuracy of the athlete selection system. Image credit: Reused with permission from (*145*). (**J**) Schematic diagram of the multichannel sensing array on a badminton racket surface. (**K**) Characterization of single electrical signals. (**L**) Characterization of multiple regional electrical signals. (**M**) Confusion matrix for predicting the batting position. Image credit: Reused with permission from (*147*). (**N**) Fabrication and structure of the E-PT yarn. (**O**) Voltage signals generated by the smart elbow pad when conducting six tennis postures. (**P**) Confusion matrix of six different tennis posture classifications. Image credit: Reused with permission from (*148*).

The TENG-based sports monitoring systems provide real-time biomechanical feedback on athletes’ movements, enabling data-driven training optimizations. By integrating ML algorithms, the self-powered systems allow coaches and athletes to adjust techniques dynamically, thereby enhancing training efficiency and effectiveness. Such systems are particularly beneficial in precision-dependent sports, such as dance and gymnastics, where accurate motion tracking is essential. TENG-based self-powered sensors capture detailed motion data, which are processed through ML models to assess movement accuracy. This facilitates early detection of improper techniques, subsequently reducing the risk of injury. Wen *et al.* (*141*) first applied recyclable flexible TENG (RF-TENG) to dance technique monitoring, developing a dance sports and injury monitoring system based on the RF-TENG sensing module (Fig. 6A). This system precisely tracked movements of knees and ankles during high-impact actions such as jumps (Fig. 6B). Using the KNN algorithm, it achieved a classification accuracy of 98.1% for various ground-jumping techniques (Fig. 6C). Similarly, Chen *et al.* (*142*) developed a lightweight, highly elastic all-aerogel TENG for self-powered intelligent fencing training, capable of detecting strike location and timing with a response time of less than 60 ms (Fig. 6, D and E). Combined with a CNN algorithm, the system classified three types of fencing strikes (thrust, miss, and whip) with 100% accuracy, supporting both training optimization and real-time competition judgment (Fig. 6F).

Beyond dance, gymnastics, and fencing, TENG-based monitoring has also been used in other sports domains for performance assessment and athlete selection (*143*, *144*). Zhu *et al*. (*145*) developed a Metaverse-based sports interactive system that incorporates TENG-based sensors for real-time interaction among users, devices, and virtual environments. A key component, the anaerobic power meter (APM) (Fig. 6G), functions as a self-powered sensing unit that collects bicycle movement data and assesses physical fitness through ML analysis (Fig. 6H). A one-dimensional CNN (1D-CNN) network was used to extract signal features, classifying athletes into four fitness levels with an accuracy of 95.37% (Fig. 6I). These findings underscore the potential of TENG technology for personalized training and systematic athlete selection, allowing coaches to develop fairer and more precise training programs (*43*, *146*).

TENG-based sports training evaluation applications have further extended to racket sports, such as badminton and tennis, where stroke precision and quality are critical performance indicators. Yuan *et al*. (*147*) designed a self-powered intelligent badminton racket using both triboelectric and piezoelectric effects (Fig. 6J). By integrating an LSTM network, the system achieved 95% classification accuracy for stroke positions (Fig. 6K) while also providing a comprehensive evaluation of stroke quality, offering athletes targeted training recommendations (Fig. 6, L and M). In tennis, Chen *et al.* (*148*) introduced a real-time training system using elastic piezoelectric-triboelectric hybrid yarn (E-PT yarn) embedded in smart elbow pads (Fig. 6N). This hybrid yarn generated enhanced energy output from triboelectric and piezoelectric signals while maintaining flexibility. Using a CNN algorithm to analyze generated voltage waveforms, this system can recognize six kinds of tennis movements with an accuracy exceeding 95%, facilitating objective, real-time evaluation of athletes’ technical movements (Fig. 6, O and P).

Overall, TENG-based sports monitoring systems demonstrate substantial potential across a wide range of athletic applications. By coupling real-time sensing capabilities with advanced ML algorithms, these systems can notably improve training precision, enhance athlete selection processes, and contribute to injury prevention. These innovations mark a transformative step toward more intelligent, efficient, and personalized sports training in the era of smart athletics.

### Sports health monitoring

**Fig. 7.**
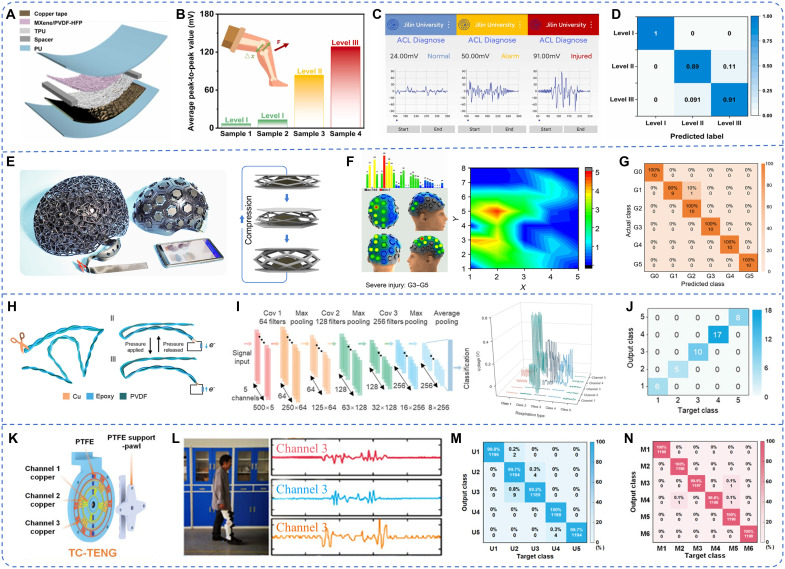
ML-assisted TENG for sports health monitoring. (**A**) MXene/PVDF-HFP composite TENG (MC-TENG) double-electrode mode structure schematic. (**B**) Summary of patient’s data and ADT schema for ACL diagnosis. (**C**) Different kinds of diagnostic results are displayed in the app. Image credit: Reused with permission from (*153*). (**D**) Confusion matrix result for the ACL diagnosis system. (**E**) Schematic of a wearable multiangle TENG (MA-TENG) sensing array and the helmet composed of it. (**F**) Visualized application screenshots and 2D cloud map distribution at G3 to G5 of head impact. (**G**) Confusion matrix of the prediction set. Image credit: Reused with permission from (*155*). (**H**) Schematic illustration of the working principle of a triboelectric sensor. (**I**) Detailed architecture of the constructed CNN model and its input data (typical respiratory signals). (**J**) Confusion matrix for the five types of respiration pattern recognition. Image credit: Reused with permission from (*158*). (**K**) Schematic of the three-channel TENG. (**L**) User output signal. (**M**) Confusion matrix result for the identity recognition for five users. (**N**) Confusion matrix for the motion recognition for six motion states. Image credit: Reused with permission from (*135*).

Sports health monitoring systems are critical for athletic training and injury prevention, enabling real-time posture correction, risk alerts, and early intervention. By continuously tracking physiological and biomechanical data, these systems help reduce injury risk, promote musculoskeletal health, and support long-term athletic performance monitoring (*149*, *150*). The integration of TENG-based sensing systems with ML enables efficient risk prediction through the analysis of physiological and motion signals, providing early warnings and personalized preventive strategies (*151*, *152*). For instance, Zhang *et al.* (*153*) developed an assembled MXene/PVDF-HFP [poly(vinylidene fluoride-*co*-hexafluoropropylene)] composite TENG (MC-TENG) for the detection of anterior cruciate ligament (ACL) injuries (Fig. 7A). The system used an ultrathin fiber network structure fabricated via electrostatic spinning, enabling precise detection of knee joint and tibial displacement under minimal pressure fluctuations (Fig. 7B). By using an SVM algorithm, the system classified ACL injury severity levels with 93.33% accuracy, demonstrating the potential for early-stage injury diagnosis (Fig. 7, C and D).

In contrast to localized injuries like ACL tears, systemic or multisite impact injuries, such as concussions, require complex and multichannel impact data analysis (*154*). Zu *et al.* (*155*) addressed this need by designing a multiangle, self-powered sensor array for real-time head impact monitoring to mitigate concussion risks. The system used multiangle TENGs (MA-TENGs) integrated with a metamaterial structure, ensuring high sensitivity and stability under repeated impact conditions (Fig. 7E). A deep CNN algorithm enabled the system to evaluate impact severity with up to 98% accuracy, providing quantitative and visualized concussion assessment and injury alerting (Fig. 7, F and G).

Effective rehabilitation after injury requires long-term monitoring of athletic performance to evaluate recovery progress and optimize intervention strategies (*156*, *157*). By leveraging the self-powered capabilities of TENGs, intelligent rehabilitation systems can collect real-time data and dynamically optimize training programs via ML algorithms, thereby enhancing recovery outcomes and minimizing the risk of secondary injuries. As shown in Fig. 7H, Fang *et al.* (*158*) developed a computational fluid dynamics–assisted mask sensor network that enables high-precision respiratory monitoring. Combined with a 1D-CNN algorithm, the system achieved 100% classification accuracy across various respiratory patterns (Fig. 7, I and J), supporting pulmonary function rehabilitation and offering potential applications in the treatment of various respiratory disorders. Similarly, Kong *et al.* (*135*) proposed a self-powered lower limb rehabilitation system based on a three-channel TENG device, capable of monitoring knee rotation angles and movement directions in real time (Fig. 7, K and L). Combined with an LSTM network for data processing, the system achieved classification accuracies of 99.68% for identity recognition and 99.96% for motion state recognition, providing robust support for rehabilitation monitoring and adaptive training adjustments (Fig. 7, M and N).

Through the integration of TENG-based health monitoring systems with ML, athletes can benefit from comprehensive injury warnings, continuous health management (*136*, *159*), accurate injury evaluation (*122*), and effective rehabilitation support (*160*). This integrated approach enhances both athletic safety and rehabilitation outcomes, highlighting the substantial value of TENG-based systems in long-term sports health surveillance.

### VR/AR sports

**Fig. 8.**
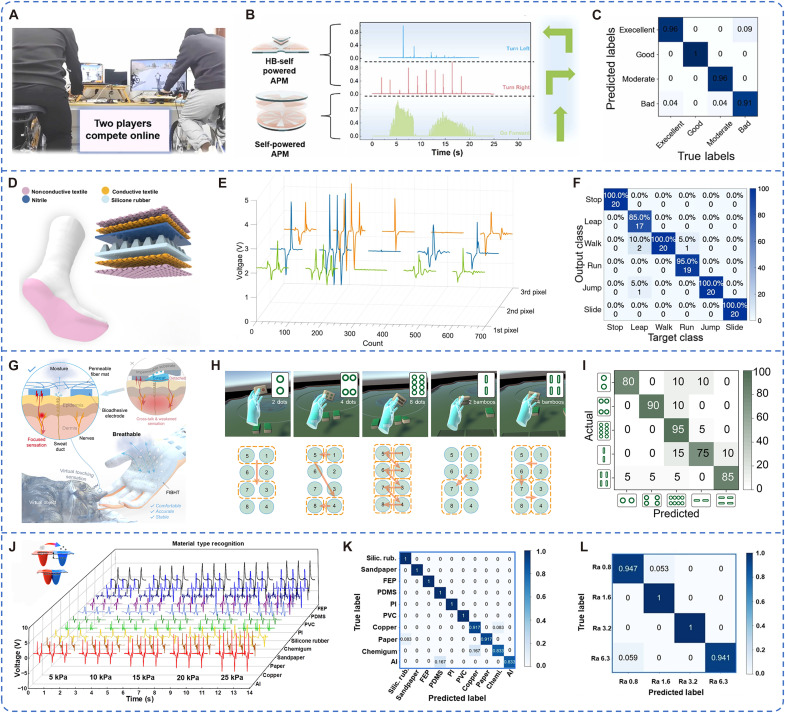
ML-assisted TENG for VR/AR sports. (**A**) VR interaction system. (**B**) Control signal generated by the self-powered APM. (**C**) Confusion matrix result for the athlete selection system. Image credit: Reused with permission from (*145*). (**D**) Textile-based TENG smart socks’ structure. (**E**) 3D plots of the DL sock outputs responding to different motions (leap, run, slide, jump, and walk). (**F**) Confusion matrix result for the recognition of six human activities. Image credit: (*48*) (CC BY; https://creativecommons.org/licenses/by/4.0/deed.en). (**G**) Conceptual illustration of the features of the FIBHT system. (**H**) Five different Mahjong tiles and the corresponding feedback patterns. (**I**) Confusion matrix result for identifying these Mahjong tiles (four volunteers, each tile for five times). Image credit: Reused with permission from (*170*). (**J**) Voltage signal when the BHES presses 10 kinds of materials under five various pressures. (**K**) Confusion matrix result for the recognition of 10 materials. (**L**) Confusion matrix result for depicting the identification accuracy of different roughness. Image credit: (*176*) (CC BY; https://creativecommons.org/licenses/by/4.0/deed.en).

VR and AR technologies are increasingly transforming the landscape of sports and fitness by offering immersive, interactive environments that bridge the gap between digital content and physical movement. VR/AR sports systems enable users to engage in realistic training simulations, gamified workouts, and real-time competitive experiences that enhance physical engagement and user motivation. However, achieving seamless interaction between human motion and the virtual environment remains a key technical challenge. The integration of TENG-based sensing technology with VR/AR sports offers a promising solution to this challenge by enabling real-time, self-powered motion tracking. These TENG-based self-powered sensing systems collect high-resolution biomechanical data during physical activity and wirelessly transmit it to the VR/AR platform, allowing for instantaneous feedback and adaptive interaction. This closed-loop system dynamically adjusts gameplay difficulty, exercise intensity, or training pacing on the basis of user performance, thereby enhancing immersion, promoting long-term adherence to physical activity, and improving the overall exercise effectiveness (*161*, *162*). Zhu *et al.* (*145*) developed a TENG-based Metaverse sports interactive system that enables remote competition between players on separate computing platforms. Integrated with a stationary bicycle, this system can capture the rider’s biomechanical signals to control in-game dynamics for immersive VR interaction (Fig. 8A). As illustrated in Fig. 8B, the APM module extracts implicit information such as peak value, contact time, and rotational speed by analyzing signal strength, amplitude variation, and frequency. By integrating with the four-layer 1D-CNN model, an accuracy of 95.37% can be achieved in identifying individual riding patterns (Fig. 8C). This innovation enhances the interactivity of sports gaming environments and promotes physical activity through engaging VR-based exercise modalities. Zu *et al.* (*163*) proposed a low-cost triboelectric smart sock that harvests biomechanical energy from low-frequency human motions (Fig. 8D). As a wearable device, it provides real-time data on user identity, physiological condition, and physical activity. Within a VR fitness game setting, the smart sock enables realistic control of virtual avatars using user-generated movements. This system can accurately identify five default motion types, including jumping, running, sliding, hopping, and walking, with a recognition accuracy of 96.67% using a 1D-CNN (Fig. 8, E and F). This integration allows players to engage in immersive gameplay while simultaneously undergoing efficient physical training.

Beyond fitness applications, the TENG technology also demonstrates substantial potential in improving the quality of life for individuals with disabilities by enhancing functional autonomy and sensory interaction. For instance, robotic limbs embedded with TENG-based sensors allow users to perform daily activities with improved control and feedback, closely mimicking the functionality of natural limbs (*164*). These sensor arrays are capable of mimicking human hand movements and recognizing materials, gestures, and locomotion patterns through wearable devices such as smart gloves and electronic skin, thereby simulating realistic haptic experiences (*165*–*169*). The integration of TENG-based sensing with DL algorithms has greatly advanced human-computer interaction in assistive technologies. Yao *et al.* (*170*) proposed a fully integrated breathable haptic textile (FIBHT), featuring an independent haptic feedback module in the palm region, fabricated from stretchable, breathable, and bioadhesive materials. Constructed from stretchable, breathable, and bioadhesive materials, the FIBHT ensures high skin permeability for effective moisture management and sustained user comfort. This system provides precise dynamic feedback with high spatiotemporal resolution and maintains excellent performance in both dry and humid environments (Fig. 8G). In VR scenarios, the FIBHT system delivers an enhanced haptic experience. For example, when a user interacts with virtual objects such as mahjong tiles, it can identify tile characteristics without visual assistance, achieving a recognition accuracy exceeding 90% (Fig. 8, H and I). This innovation offers a promising pathway for developing immersive, accessible, and functionally supportive environments tailored to individuals with physical impairments.

To further enhance the autonomy of individuals with disabilities in both athletic performance and daily activities, the TENG technology has also been used in the development of advanced electronic skin (*166*, *171*–*175*). Tao *et al.* (*176*) introduced a biomimetic, ultrasensitive, and multifunctional hydrogel-based electronic skin (BHES) that interfaces with the external environment by integrating signal acquisition and processing circuits for complex tactile tasks. When interacting with various materials or textured surfaces, the BHES produces distinct electrical signals corresponding to surface characteristics. Leveraging AI algorithms, this system can accurately distinguish between subtle differences in texture and material composition (Fig. 8J). It achieved recognition accuracies of 95.00% for 10 types of materials and 97.20% for four different surface roughness levels (Fig. 8, K and L). This capability enhances environmental perception for prosthesis users, thereby improving their quality of life and enabling various applications in sports technology (*177*, *178*).

In summary, the TENG technology exhibits substantial potential for advancing human-computer interaction, particularly when integrated with DL algorithms. Whether applied to real-time motion feedback in VR environments or to assistive technologies for individuals with disabilities, this synergy enhances interaction accuracy, responsiveness, and user experience. Such innovations are driving progress in rehabilitation, immersive gaming, and wearable sensing systems for smart and inclusive technology development.

## DISCUSSION AND OUTLOOK

Intelligent sports facilities and wearable equipment are increasingly adopted in the sports industry, driving the trend toward digitalization and intelligent systems (*179*–*181*). Maintenance-free, sustainable, and highly sensitive sensors are essential for acquiring and analyzing large-scale, real-time motion data. In this review, we have summarized recent advancements in TENG technology for constructing self-powered intelligent sports systems, with a particular emphasis on its integration with ML. The integration of TENGs with ML presents a promising approach for enhancing intelligent sports applications, especially in the domains of real-time motion sensing, adaptive feedback, and data-driven decision-making. By leveraging the flexible, self-powered, and high-sensitivity features of TENGs alongside the data processing and pattern recognition capabilities of ML, this integrated system supports advanced use cases such as performance evaluation, sports health monitoring, and VR interaction. Interdisciplinary studies have already demonstrated encouraging outcomes in both controlled laboratory and semipractical environments.

**Fig. 9.**
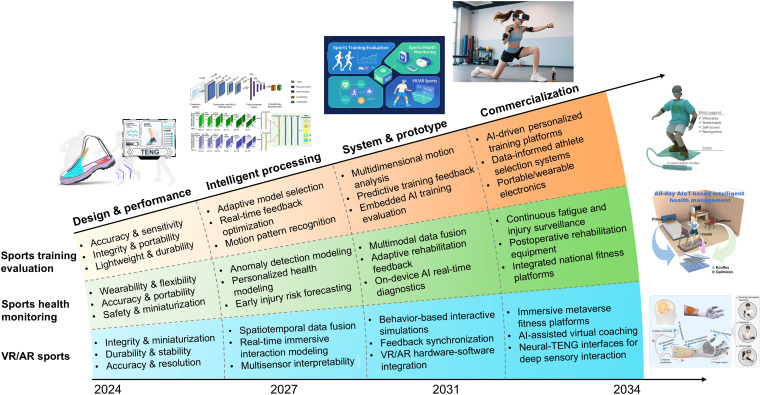
Roadmap of MTIS development from 2024 to 2034. Image credits: Reused with permission from: (*85*, *163*, *195*) and from (*159*, *167*, *196*) (CC BY; https://creativecommons.org/licenses/by/4.0/deed.en).

On the basis of the three major application domains, Fig. 9 illustrates a strategic development roadmap for MTIS, segmented into four progressive stages spanning from 2024 to 2034. This roadmap identifies critical research priorities and technological milestones that must be addressed to achieve widespread, impactful adoption of MTIS in real-world applications. In the initial stage, efforts are centered on innovative structural design and performance optimization of TENG devices tailored for domain-specific sensing requirements. The second stage emphasizes the selection and customization of ML algorithms to maximize the effectiveness of motion data processing and visualization for each specialized application. Stage three focuses on critical system integration, prototype validation, and product development processes. The final stage targets full-scale commercialization, addressing challenges such as mass production, regulatory compliance, cost-effectiveness, and standardized performance evaluation. Overall, this roadmap outlines a coherent, interdisciplinary pathway for transforming MTIS into a mature and widely adopted intelligent sports technology.

Despite notable advancements, the integration of TENG and ML in intelligent sports remains in its early stages, facing multiple technical and systemic challenges. To unlock their full potential and achieve widespread adoption, this field must address several core areas: device robustness, intelligent signal processing, standardized development pathways, diversified applications, user-centric design, and sustainable commercialization.

1) Enhancing TENG performance and data quality. In practical applications, TENG-based intelligent sports systems face multiple external environmental challenges such as wind, humidity, and temperature. Sweat and salt released during user exercise can also infiltrate and contaminate TENG devices, leading to instability and performance degradation. That all remains a critical barrier for outdoor and long-term use. To address these issues, improving material selection and structural design can further enhance TENG output, durability, and data quality (*182*–*186*). Moreover, developing advanced packaging technologies can prevent moisture intrusion without noticeably impairing output performance (*187*).

2) Optimizing ML for TENG-based intelligent systems. To fully leverage the synergy between TENG and ML, it is essential to develop ML algorithms tailored specifically to TENG devices and the demands of different sports applications. Custom-designed models can enhance motion recognition accuracy, system responsiveness, and adaptability. Future research should also focus on the seamless integration of these intelligent algorithms with hardware, establishing fully integrated, self-powered sensing systems for personalized motion monitoring, adaptive rehabilitation training, and intuitive user interaction. Moreover, the incorporation of AI chips with on-device training capabilities and low power consumption will be crucial, enabling real-time learning and adaptive processing in mobile and wearable environments. Addressing the variability in individual users will be critical for improving model generalization. By enhancing the interpretability and robustness of ML models in handling TENG-generated signals, TENG-based intelligent sports systems can become more personalized, reliable, and efficient.

3) Standardization and cross-disciplinary collaboration. Despite rapid progress, the integration of TENG and ML technologies in intelligent sports remains in the early stages, lacking standardized performance metrics, data collection protocols, and model evaluation criteria. Establishing unified technical standards will improve system comparability, reproducibility, and scalability while also facilitating broader adoption across the sports industry. In addition, interdisciplinary integration such as materials science, electronic information, and computer science can drive innovation throughout the development chain. Joint efforts in designing high-performance TENG materials, optimizing sensor architectures, advancing learning algorithms, and collecting diverse, high-quality sports datasets will accelerate the translation of lab-scale prototypes into practical applications.

4) Expanding application scenarios and sport types. Current TENG-based systems are primarily focused on specific motion-intensive or precision-demanding sports such as running, ball games, and dance. Future efforts should explore applications in a wider range of sports, including team-based games, aquatic sports, and adaptive sports for individuals with disabilities. This expansion requires the customization of sensor design and data analysis models to accommodate distinct biomechanical patterns and performance indicators across different sports. In addition, large-scale deployment in real-world contexts, such as training facilities, rehabilitation centers, and competitive arenas, will be essential to validate, refine, and enhance the functionality and robustness of TENG-based systems under diverse use conditions.

5) Enhancing user experience and feedback mechanisms. User-centric design is critical for the widespread adoption of TENG-based intelligent sports systems. Real-time, intuitive, and multimodal feedback interfaces (such as haptic responses, visual dashboards, and audio cues) can substantially improve user engagement and system usability. These features can be integrated into VR/AR platforms or mobile applications for immersive and responsive interaction. Personalized feedback, tailored to users’ physical conditions, performance goals, and training progress, can further enhance motivation and effectiveness. Moreover, incorporating gamification elements and social interaction features may foster a sense of achievement and community, particularly among amateur athletes and younger users, contributing to long-term adherence and broader market appeal.

6) Accelerating commercialization and policy-driven development. TENG-based intelligent sports systems offer substantial commercial value in wearable equipment, sports facilities, digital health, and VR/AR applications. However, commercialization is contingent on addressing challenges such as manufacturing scalability, algorithm robustness, and cost reduction. To accelerate industry growth and translation to practice, strategic initiatives are needed. Government funding for foundational research, pilot programs in public sports facilities, and policy incentives for innovation can substantially reduce barriers to entry. In parallel, public-private partnerships, with athletic brands, health care providers, and tech companies, can expedite development cycles and market readiness. Moreover, regulatory pathways must be clarified, especially for health care–related applications, where compliance with medical device standards is essential. Coordinated efforts across academia, industry, and government will play a pivotal role in shaping an ecosystem conducive to innovation and sustainable growth.
