# Supplementary material for: Machine learning–assisted triboelectric nanogenerator technology for intelligent sports
Source: Sci Adv. 2025 Oct 1;11(40):eadz3515. doi: 10.1126/sciadv.adz3515 (PMC12487898; doi:10.1126/sciadv.adz3515)
Supplement: Supplementary file 1 — Fig. S1 Table S1 [file sciadv.adz3515_sm.pdf]

Science Advances
AAAS

Supplementary Materials for

Machine learning–assisted triboelectric nanogenerator technology for intelligent sports

Minglan Ji *et al.*

Corresponding author: Jianjun Luo, luojianjun@binn.cas.cn; Haibo Zhou, haibo.zhou@jnu.edu.cn; Zhong Lin Wang, zhong.wang@mse.gatech.edu

Sci. Adv. **11**, eadz3515 (2025)
DOI: 10.1126/sciadv.adz3515

This PDF file includes:

Fig. S1
Table S1

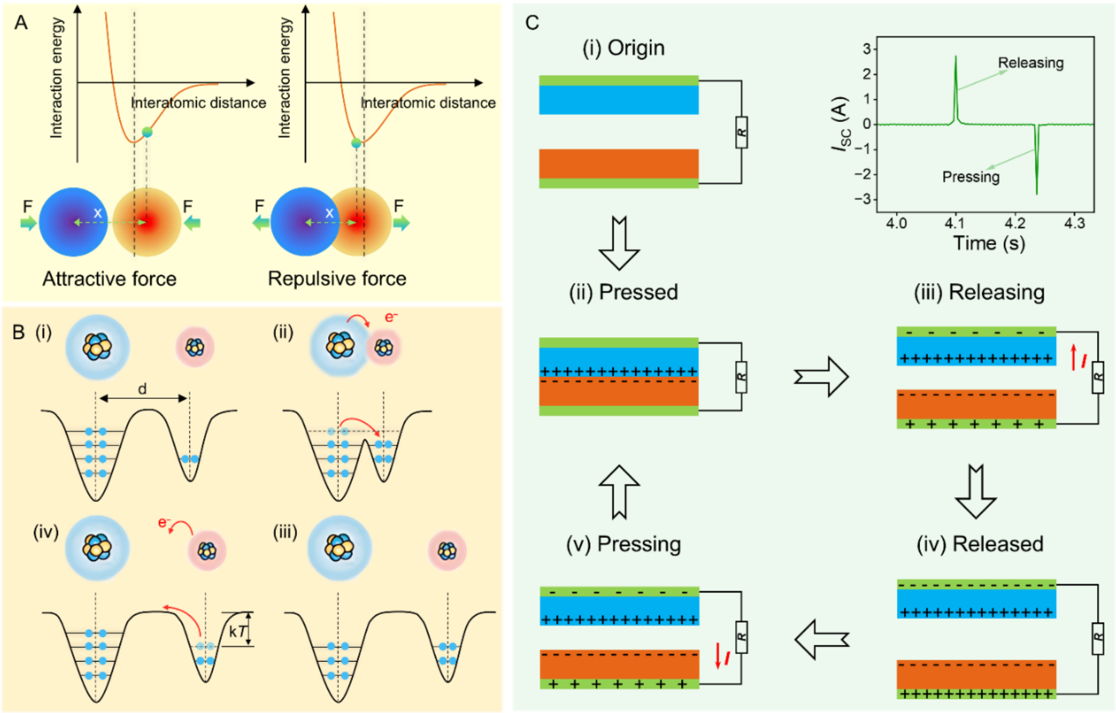

Fig. S1. CE Mechanism of TENG. (A,B) The overlapped electron-cloud model (A Interatomic interaction potential between two atoms. B Schematic of the electron cloud and potential energy well model of two atoms belonging to two materials.). **(C)** Working principle of the contact-separation mode TENG.

Table S1 Comparison of sensing technologies in intelligent sports based on resistive, capacitive, electromagnetic, optical, triboelectric, piezoelectric, and pyroelectric mechanisms.

	Mechanism	Advantages	Disadvantages
Resistive	Resistive transduction	Low cost, simple structure	Low sensitivity, susceptible to temperature
Capacitive	Capacitive transduction	High sensitivity, low power consumption	High cost, weak environmental adaptability
Electromagnetic	Electromagnetic induction	Long service life, fast response, high efficiency at high frequency	Bulky structure, high cost, susceptible to magnetic interference
Optical	Optical modulation	High sensitivity, high environmental adaptability	High cost, complex signal demodulation
Triboelectric	Contact electrification & electrostatic induction	Self-powered, simple structure, light weight, low cost, diverse choice of materials, high efficiency at low frequency	Limited durability
Piezoelectric	Piezoelectric effect & electrostatic induction	Self-powered, high sensitivity, fast response	Low output, complex fabrication
Pyroelectric	Pyroelectric effect	Self-powered, sensitive to temperature fluctuation	Low output, complex fabrication
